# Do executive functions and processing speed mediate the relationship between socioeconomic status and educational achievement? Analysis of an observational birth cohort study

**DOI:** 10.1186/s40359-024-02243-1

**Published:** 2024-12-18

**Authors:** Kate E. Mooney, Rachael W. Cheung, Sarah L. Blower, Richard J. Allen, Amanda Waterman

**Affiliations:** 1 Department of Health Sciences, University of York, York, England; 2 Better Start Bradford Innovation Hub, Bradford Institute for Health Research, Bradford, England; 3 Born in Bradford Centre for Health Data Science, Bradford Institute for Health Research, Bradford, England; 4 School of Psychology, University of Leeds, Leeds, England; 5 Centre for Applied Education Research, Bradford Institute for Health Research, Bradford, England

**Keywords:** Socioeconomic status, Executive function, Educational achievement, Child development

## Abstract

**Background:**

There are large and persistent social inequalities in children’s educational attainment, with children from more socioeconomically disadvantaged families consistently having lower attainment. Despite this being widely reported, the mechanisms underlying the association between socioeconomic disadvantage and educational attainment are not well understood. It is important to understand the potential mechanisms by which socioeconomic disadvantage may impede on educational outcomes, as this knowledge could then be used to help target possible interventions to improve educational outcomes for socioeconomically disadvantaged children. Children’s executive functions (including working memory and inhibition) and processing speed abilities may underlie these inequalities, however, the previous literature regarding this is limited. This study examined longitudinal mediating mechanisms between socioeconomic status (SES) and educational achievement, using a socioeconomically deprived and ethnically diverse cohort.

**Methods:**

Data from the Born in Bradford longitudinal cohort study was analysed using Structural Equation Modelling (*n* = 4201; 28% White British, 56% Pakistani heritage, 16% Other; 54% Female). SES was measured before birth, executive functions and processing speed were measured in middle childhood (M_age_=8.45 years), and educational achievement was obtained through educational records (M_age_=10.85 years). All models adjusted for child gender, age, language ability, ethnicity, and mother immigration status.

**Results:**

Executive functions significantly mediated the association between SES and educational achievement (B = 0.109), whilst processing speed did not. Examination of executive function components revealed that working memory significantly mediated the associated between SES and educational achievement (B = 0.100), whilst inhibition did not. Working memory appeared to account for a large proportion (39%) of the total effect of SES on educational achievement.

**Conclusions:**

These results, and the theoretical mechanisms linking working memory to educational achievement, both indicate the importance of finding ways to support children with working memory difficulties in the classroom. This is an important avenue for future research and may be useful for closing the socioeconomic gap in educational achievement.

**Supplementary Information:**

The online version contains supplementary material available at 10.1186/s40359-024-02243-1.

## Background

Large and persistent inequalities in children’s educational outcomes have consistently been demonstrated by socioeconomic status (SES); sometimes referred to as the educational achievement gap [1]. A meta-analytic review of over 100,000 children living in the USA found a clear positive relationship between socioeconomic status and educational outcomes [2]. In the UK, only 63% of children living in the most deprived areas achieve expected levels in national Reading, Writing, and Mathematics educational assessments, whereas 86% of children living in the most affluent areas achieve expected levels [3]. Failure to achieve these core qualifications significantly hinders a child’s progression into further employment or further education [4]. The cumulative impact of not achieving educational qualifications thus leads to long-term challenges not only for the individual, but also for societies as a whole [5].

It is important to understand the potential mechanisms by which socioeconomic disadvantage may impede on successful educational outcomes, as this knowledge can be used to help target possible interventions to improve educational outcomes for socioeconomically disadvantaged children. Researchers have started to examine the longitudinal pathways between socioeconomic inequality, potential mediating factors, and children’s educational achievement. However, studies tend to focus on one potential mediator at a time (e.g., a composite of executive functions [6, 7]). Whilst it is beneficial to establish the significance of individual mediators, more information can be gained from comparing multiple mediating pathways simultaneously, as these likely have related, but distinct, effects on educational attainment. In this study, we compare different types of executive functions, and processing speed, as separate mediators in the association between socioeconomic status and children’s educational achievement. By doing so, we allow a richer insight into the underlying mechanisms between SES and educational achievement, which in turn could help inform best use of targeted interventions to ameliorate the potential effects of socioeconomic disadvantage on educational achievement.

### Executive functions

One way in which SES may influence children’s educational outcomes is via executive functions. Executive function (EF) is an umbrella term that encompasses the processes responsible for purposeful, goal-directed behaviour [8]. In this study we examine two core components of EF that have been identified in children [9]: (1) Working Memory (WM), a limited capacity system that allows the storage and manipulation of information over short time periods [10], and (2) Inhibition, which can be defined as the ability to deliberately inhibit dominant or automatic responses [11]. Whilst there is some debate in the literature about the exact nature of EF in childhood [12], there is evidence showing that the structure of EF is probably best represented by a two-factor model during childhood, where WM is separable from inhibition between the ages of 5–10 years [13], compared with a three-factor ‘adult’ model of EF which emerges after the age of 11 years, containing shifting, inhibition, and WM [14].

Prior to establishing mediating mechanisms, it is fundamental to first establish direct associations between SES and EF, and between EF and educational outcomes. Previous studies have shown that higher SES is associated with higher scores on EF tasks [15, 16]. This could be due to environmental factors linked to low SES, including heightened stress and lower nutrition, both of which may negatively impact the development of brain areas responsible for EF [17, 18]. For example, one study has found maternal psychological distress to be a consistent mediator between SES and EF [19].

However, some researchers have argued that it is more likely to be the advantages of socioeconomic wealth, compared with the harmful effects of low SES (e.g. via stress), that drive the socioeconomic differences in children’s outcomes [20]. Socioeconomic resources can provide additional opportunities, including a more enriched language and home environment and a greater education quality, resulting in positive changes in children’s brain development, and EF [20]. One study showed that neighbourhood SES was associated with WM via greater activation in specific brain areas, supporting the idea that the broader local environment can support a child’s cognitive development through access to community and educational resources outside of the home [21]. It is important to note that whatever the reasons may be for socioeconomic differences in children’s outcomes, we do not take a ‘deficit-based’ lens which discusses children as simply lacking skills [22]. Instead, we stress the importance of an approach that considers how families at the intersection of various levels of socioeconomic disadvantage are influenced by many sociopolitical and environmental determinants that interact with one another [22, 23].

It has also been shown that higher scores on EF tasks measured in childhood are associated with better educational achievement both in childhood [24, 25], and in adulthood [26]. EF underpins many abilities required in a classroom setting: maintaining and shifting attention during a lesson, remembering classroom rules, and using planning to solve problems effectively [6]. More recently, a few studies have found that EF mediates the association between SES and educational achievement. Two of these studies looked specifically at mathematical skills, and found that an ‘overall’ EF score (a composite of two or more tasks relating to different EFs) mediated the association [15, 27]. Two other studies found that composite EF mediated the association between SES and broader educational achievement [6, 28]. Whilst it is useful to establish that EF mediates the association between SES and educational achievement, it does not establish *which* of the components of EF may be most important in this association, or whether they are equally important. It is possible that certain components of EF are providing the strongest underlying pathways in the association between SES, EF, and educational achievement, and that other components of EF are ‘masking’ or ‘weakening’ the associations.

To the best of our knowledge, four studies have specifically investigated the role that individual components of EF play in mediating the association between SES and educational achievement. Three of these studies found that WM significantly mediated the association, in comparison to other abilities (including verbal ability, cognitive flexibility, inhibition, and attentional control) which did not significantly mediate the association, either at all, or as strongly. Two of these were longitudinal studies which followed children from ages 8 to 13 years [29], and 1-month-old to 8 years [30], and one was cross-sectional, with children aged 8-years-old [31]. However, the fourth demonstrated contradicting results in a cross-sectional study with 3-4-year-olds, finding a relationship between SES and educational achievement via inhibitory control, but not via WM [32].

The current study will build on the findings of these studies in important ways. Only two of the previous studies have used longitudinal data, whereas the other two have used cross-sectional data [31, 32]. In comparison to more appropriate longitudinal data, mediation analyses of cross-sectional data can lead to different and potentially inaccurate estimates regarding the mediation process under study. Mediation is a process that unfolds over time, therefore it is essential that a temporal sequence is apparent in the data, where the independent variable precedes the mediator, and the mediator precedes the outcome [33].

Next, related to the above issue, it is important to consider the timing by which these processes unfold. Previous research has established the presence of cross-sectional associations at 4 years-old and 8-years-old [31, 32], across the early years period between 1 month and 5-years-old [30], and across middle childhood between age 9 years and 13 years [29]. However, it is important to examine whether SES measured during pregnancy and early life has longstanding associations with later outcomes into the middle childhood period, particularly since children are most susceptible to their environments in the earliest years of their lives [18, 34]. This information could be used to target interventions which could mitigate the impacts of early socioeconomic disadvantage.

Further, most of these studies have relied upon tests of educational abilities that are not part of children’s educational records, limiting their generalisability to having real world implications for children [30–32]. It is crucial to build an understanding of the impact of SES and EF on children’s performance on the examinations and tests that become part of their educational record and which most children routinely undergo, for example, the Scholastic Aptitude Test (SAT) in the US, or Key Stage Tests in the UK. These tests influence a child’s path through education and predict life outcomes in terms of their socioeconomic mobility, as a child’s educational achievement determines their future chances of obtaining further qualifications in higher education and influences their options for future employment [4]. It is therefore important to use these types of tests as the outcome variable to understand fully how different aspects of EF mediate the association between SES and real-world educational achievement.

Finally, our understanding of whether these associations are generalisable to different ethnic groups is limited. ‘Ethnicity’ as a construct encompasses shared descent, heritage and culture, and often includes shared religion, tradition and language [35]. In England, ethnic minority groups tend to experience higher levels of socioeconomic disadvantage [35]. Ethnicity can be associated with SES [36], executive functions [16, 37], and educational achievement [3], therefore a lack of consideration of ethnicity may potentially lead to a biased understanding of these associations.

A previous systematic review and meta-analysis on ethnic group differences in executive functions tasks in US samples found large absolute differences between ‘White’ ethnic groups and minority ethnic groups, and medium sized differences between ethnic minority groups. ‘White’ ethnic groups had higher scores than ethnic minority groups overall, and the authors conclude that this could be due to stereotype threat, racism, race-based social stress, linguistic ability, and/or acculturation [37]. Further, many tasks of EF were normed and developed with White children, which brings into question whether they are as valid a measure for ethnic minority children [22]. Existing research on the sample used in the present study found differences in WM scores between ‘White’ and ethnic minority groups, where many of the ethnic minority groups had higher scores on WM tasks than the White British group [16], suggesting that these relationships may differ by context and country.

The consideration of ethnicity is also important for variation in educational achievement. In England, pupils belonging to ethnic minority groups make up 31.8% of the total school population, with pupils of Pakistani heritage being the largest single ethnic minority group, at 4% of the total school population [3]. National data in England indicates that most ethnic minority groups tend to have higher levels of educational achievement than White British pupils at age 16. However, there are intersectional inequalities by both ethnicity and SES, with White British and Black Caribbean/Mixed White & Black Caribbean students from low SES backgrounds having the lowest educational achievement nationally in England [38, 39]. It is therefore possible that research relating EF to educational achievement does not represent a wider population, since it so often relies upon ‘White’ ethnic groups [22], and frequently does not consider the role of ethnicity in these associations. Again, any differences in EF and educational achievement by ethnic group are likely a result of the complex intersection between socioeconomic, sociopolitical, and other environmental experiences [22, 23].

### Processing speed

Another ability through which socioeconomic status may influence children’s educational achievement is via processing speed, which relates to how quickly children process information, and is normally measured using a reaction-time task [40, 41]. It has been proposed that individual differences in EF may be driven by processing speed, and that inclusion of processing speed measures may further explain links between EF and educational achievement [40].

Previous studies have found that processing speed has distinct associations with children’s educational achievement that are separable from the association between WM and educational achievement. Processing speed and higher WM scores predicted higher overall achievement in a sample of 65 children aged 9–10 years [41]. In contrast, faster processing speed at 5 years related to higher math achievement at 6 years, but WM skills did not [42].

To the best of our knowledge, only two studies have investigated the relation between childhood socioeconomic status and processing speed. Socioeconomic status was related to better performance on a perceptual processing speed task in 7–11-year-olds, a relationship that was consistent across both White and African American children [43]. A similar result was found with an ethnically diverse sample of 4-5-year-olds, but where processing speed was measured via reaction times in response to a WM task [44]. As with the potential mechanisms behind SES and EF, this association may occur through the negative impact of low SES related factors such as stress and nutrition [17–19], or through the enriched opportunities gained by higher SES [20, 21]. However, and with particular relevance to the current study, no studies have tested the mediation between socioeconomic status and educational achievement via processing speed.

### Study context and objectives

In this study we tested the different contributions of EF and processing speed abilities during middle childhood, to the relations between early life SES and educational achievement at age 10-years-old. The study uses data from a longitudinal cohort study based in Bradford, England. Levels of child poverty in Bradford are amongst some of the highest in England, with 39% of children living in relative poverty after housing costs in 2020/21 [45]. Bradford is an ethnically diverse city, with 57% of school pupils belonging to an ethnic minority, and 63% of those pupils being Pakistani heritage [46]. Since ethnic group may be associated with differences in both EF [16, 37], and educational achievement [3], we included a diverse range of ethnic groups in our analyses, and adjusted our models for the potential effect of ethnicity.

Based on previous research we expect that the association between SES and educational achievement will be mediated by overall EF. Given the lack of research looking at processing speed as a mediating factor we did not have any predictions about whether processing speed would be a stronger or weaker mediating factor after controlling for mediation via EF. Further, we tested the contributions of the two core EF components (WM and Inhibition) to the relations between SES and educational achievement. Whilst, on balance, previous research suggests that WM may be a stronger mediator when compared to other executive functions, this previous research has limitations, and the findings have been contradictory [32]. We therefore anticipate that the association between SES and educational achievement is likely to be more strongly mediated by WM than by inhibition.

## Methods

### Design

This is a secondary analyses of an observational cohort study. We follow the Strengthening the Reporting of Observational Studies in Epidemiology (STROBE) Statement reporting guidelines for reporting in this study (Supporting Document, Attachment A). The study analyses were preregistered on the Open Science Framework (OSF) at https://osf.io/p7z6q.

### Setting

The data source is the longitudinal cohort study, Born in Bradford (BiB), which recruited pregnant mothers between March 2007 and December 2010. Bradford is the sixth-largest metropolitan district in England, with 64% of the population identifying as White British and 20% identifying as Pakistani heritage [47]. Bradford is an area with high levels of child poverty overall (Barnes, 2022), with the district being the 13th most deprived local authority of 326 in England (City of Bradford Council, 2019, 2021). All babies born to mothers at the main Bradford hospital were eligible to participate and more than 80% of women invited agreed to participate. The cohort comprises of 12,453 mothers, 13,776 pregnancies and 3,448 fathers. At recruitment, the two largest ethnic groups in the sample were Pakistani heritage (45%) and White British (40%) [36, 48].

Mothers completed a baseline questionnaire during pregnancy and reported information on family demographics and several indicators of socioeconomic position. Large-scale data collection took place between 2016 and 2019, including assessments of child physical activity, wellbeing, and cognition. This data collection period is referred to as the ‘Primary School Years’ wave. Data were collected across three academic school years (Year 3–6, covering ages 7–11 years) in 90 schools that had high numbers of BiB children. Researchers tested whole classes of children at a time, including both children who were and were not part of the BiB study. The Primary School Years wave therefore contains data from 15,820 children, of which 9604 are participating in the BiB cohort study [49].

### Measured variables

**Fig. 1:**
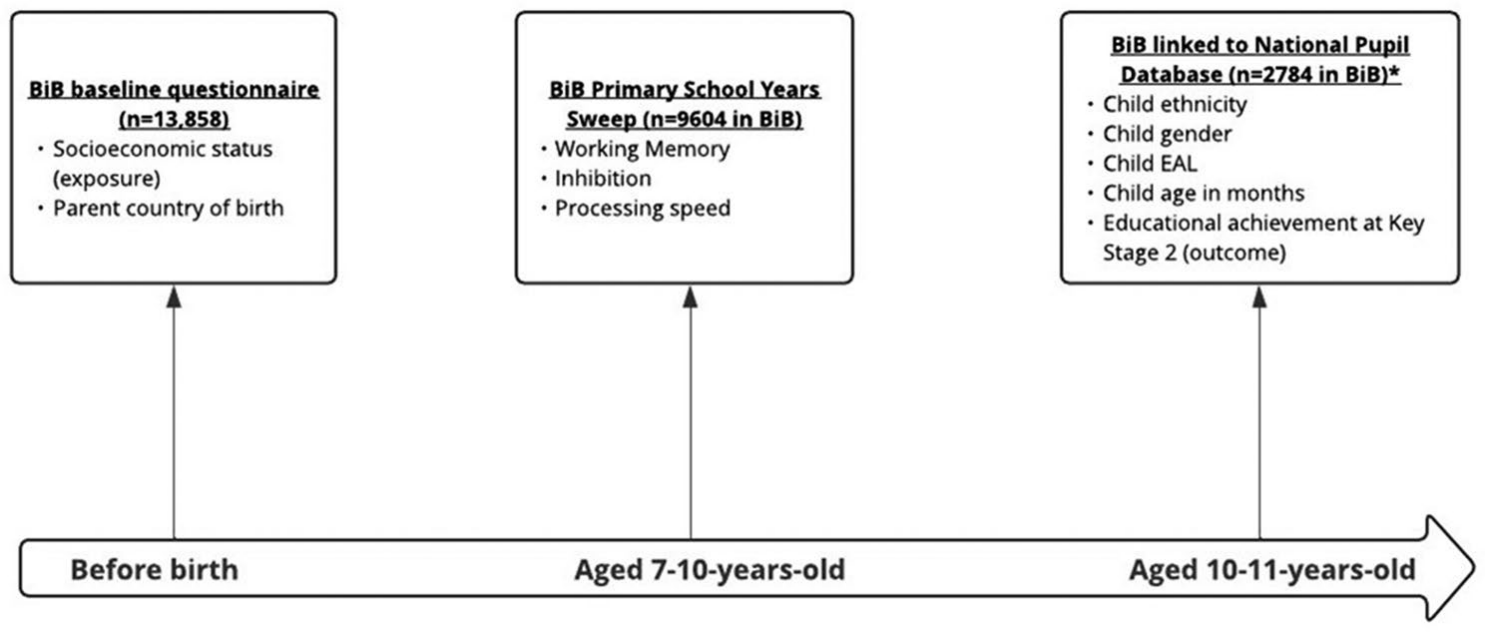
Overview of measures variables and data sources. *Note: Not all children had linked data for the Key Stage 2 educational achievement outcome, as not all BiB children had reached this age at the time of data curation (the total number with Key Stage 2 outcomes was correct on 14/08/2024). Abbreviation: EAL = English as an Additional Language

**Fig. 2:**
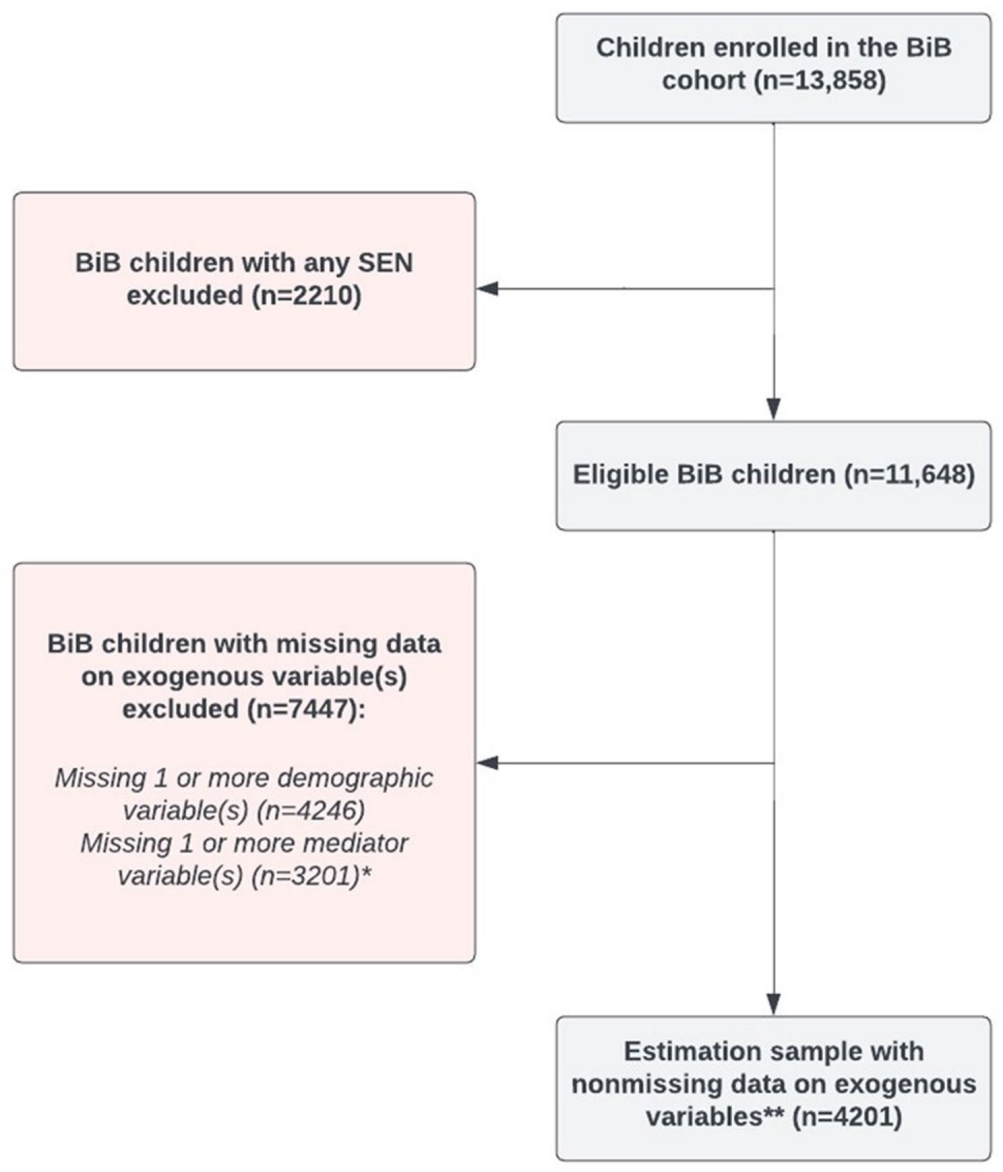
Availability of measures in study sample. Note: *As ‘age at time of executive function/processing speed measure’ was an exogenous variable, children were excluded if they did not have available data on the mediator variables. **Exogenous variables were: (1) mother immigration status (2), child ethnicity (3), child age at time of EF/processing speed (4), child gender (5), SES, and (6) child EAL. Abbreviations: BiB = Born in Bradford; EAL = English as an Additional Language; SEN = Special Educational Needs; SES = Socioeconomic Status

Figure 1 provides an overview of the measured variables within the data sources used, and the timepoints at which they were collected. Though both collected at 10-years-old, all BiB Primary School Years data were collected before educational achievement data for each child. The sample sizes provided here are available data for the whole wave of children, and the sample size for our estimation sample are provided in Fig. 2.

#### Exposure

*Socioeconomic status.* Since SES is a multidimensional concept that includes both resource (e.g. income, wealth) and prestige (e.g. education, occupation, societal position) components [50], it is therefore important to capture these different components to fully understand the potential influence on different outcomes. It is also important to carefully consider which measurements of SES to use for different ethnic groups as the meaning of socioeconomic constructs will vary across cultures and context. For example, educational achievement may have different meanings for different ethnic groups, particularly if it has been received in different countries [51].

In BiB, a comprehensive socioeconomic measure has been developed and validated for use across the diverse ethnic groups within the cohort (for further details see [36]). The measure has been previously used to investigate the direct effects of socioeconomic status on child cognitive and health outcomes [16, 52], or control for socioeconomic status when investigating effects of different environmental variables on child health outcomes [53]. It aggregates detailed socioeconomic information across 19 different variables from the maternal baseline questionnaire during pregnancy and includes (but is not limited to) employment and education per parent, subjective financial indicators, receipt of means-tested benefits, ability to keep up with bills, housing tenure, and affordability of basic living needs. These were used to provide a more detailed description of the socioeconomic profile of a multi-ethnic population than more traditional indicators of SES, such as education or occupation. Education can have different meanings for multi-ethnic populations if, for example, a qualification was received in a different country [51]. The measure used Latent Class Analysis to group mother’s socioeconomic position into 5 distinct categories, where 1 = least deprived (an affluent and well educated group), 2 = employed, not materially deprived (a group who were working but not materially deprived), 3 = employed, no access to money (a group who were working but were materially deprived), 3 = benefits, but coping (a group with low levels of deprivation and high uptake of benefits), and 5 = most deprived (a group with high uptake of benefits and high material deprivation) [36]. The LCA estimated groupings using posterior probabilities of belonging to each group. For a description of participant characteristics within these groups, please see the online material (Supporting Document, Attachment B). Given this variable has five categories it was estimated using a multivariate normal distribution in the following analyses (i.e., maximum likelihood estimation) [54].

*Mediators.* All tasks were administered at 7–10 years (M_age_ =8.42, SD = 0.66) as part of the BiB Primary School Years data collection. For the children in our estimation sample, all tasks were administered prior to Key Stage 1 assessments (the outcome). Assessments included a battery of five cognitive tasks (three Working Memory tasks, Inhibition, and Processing Speed). Tasks were administered by researchers in the classroom, presented in the same order via a tablet computer (Lenova ThinkPad Helix type 20CG or 20CH) with instructions delivered by headphones, and finger touch used for response input. Accuracy and reaction time (RT) data were collected. Extreme scores on cognitive tasks were retained in models, as these were likely ‘interesting’ outliers which represent children’s true extreme scores, rather than ‘error’ outliers which represent data entry errors [55]. For further details see Hill et al. (2020).

Working Memory. Participants completed three WM tasks (1). Forwards Digit Recall (FDR): participants heard sequences of digits and had to recall them in the same order they were presented. Sequence length increased from length 3 to length 6, with four trials at each length (2). Corsi: participants saw nine randomly arranged blocks on the tablet screen, which lit up one at a time in a particular spatial sequence, and the participant had to recall the spatial sequence in the same order it was presented. Sequence length increased from length 3 to length 6, with four trials at each length (3). Backwards Digit Recall (BDR): participants heard sequences of digits and had to recall them in reverse order. Sequence length increased from length 2 to length 5, with four trials at each length. The outcome variable for each WM task was mean proportion correct (range 0-100) and these scores were used as indicators of a latent variable for WM. We used confirmatory factor analysis (CFA) in *R* using the package *lavaan* [56] to fit this latent WM variable. All factor loadings were > 0.30 and significantly loaded onto the latent variable (*p* < .05, CFA fit indices: χ2(0) = 0.00, CFI = 1.00, RMSEA = 0.00, SRMR = 0.00).

*Inhibition* (Flanker task). Children were presented with a line of five arrows and were required to identify the direction of the middle arrow. There were 40 test trials. For half of the trials (congruent) all the arrows pointed in the same direction, and for the other half (incongruent) the middle arrow pointed in the opposite direction from the other arrows, with these trials randomly intermixed. The Flanker task is a widely used measure of response inhibition as it assesses the subject’s ability to suppress responses that are inappropriate, as inhibition is required to refrain from consistently identifying arrows as pointing in the right direction [57]. Children were asked to answer as quickly and accurately as possible. The outcome variable was mean (Reaction Time (RT) to congruent trials – RT to incongruent trials).

Processing speed. Children were presented with a random number of red circles, red triangles, and blue circles, and asked to identify how many red circles were present on the screen by tapping a box located at the bottom of the screen with the correct number. There were 18 trials in total, and children were asked to carry out each trial as quickly and accurately as possible. The outcome variable was mean RT to correct trials.

Executive function. We modelled executive function as a latent variable using CFA, with raw scores from WM and inhibition tasks as the indicator variables. As latent variables are unobserved and therefore have no definite metric scale, the loading of the first indicator variable of the latent variable is constrained to 1, establishing the scale measurement and allowing a combination of indicator variables with different scales to be used under one latent variable [58]. All factor loadings were > 0.30 and significantly loaded onto the latent variable (*p* < .05, CFA fit indices: χ2 (2) = 132.694, CFI = 0.959, RMSEA = 0.114, SRMR = 0.033)

#### Outcome

Educational achievement: The Key Stage 2 Assessment is a statutory, national, standardised test conducted under exam conditions and set by the UK Standards and Testing Agency. It is completed towards the end of Year 6 at school (age 10–11 years). There are continuous scaled scores for mathematics, reading, and grammar/punctuation/spelling that range between 80 and 120. These were all used to create a latent variable of ‘educational achievement’. All factor loadings were > 0.30 and significantly loaded onto the latent variable (*p* < .05, CFA fit indices: χ2(0) = 0.00, CFI = 1.00, RMSEA = 0.00, SRMR = 0.00). As the BiB cohort was recruited between 2007 and 2010, only a subset of the sample have linked and available Key Stage 2 outcome data (as not all children were old enough at the time the data curation was completed).

#### Covariates included in all models

Variables were selected to be included in the model if they were thought to be a confounder, i.e., they were considered to be potentially causally associated with both SES and educational achievement (Immigration status, Ethnicity, English as an Additional Language), or they were covariates considered to be potentially causally associated with just the outcome (Age, Gender).

*Mother’s country of birth.* Mother’s country of birth was collected in the baseline questionnaire and was coded as 0 = Born in United Kingdom, 1 = Born outside of United Kingdom.

*Child ethnicity.* Child ethnicity was obtained through the national education records that are updated annually. We originally planned to adjust for ethnicity using three categories (White British, Pakistani heritage, and Other), however, the ‘Other’ group (*n* = 645) was too small to estimate within the model, in comparison to the White British (*n* = 1174) and Pakistani heritage (*n* = 2382) groups. This is because variability within the exogenous variables (e.g. SES, English as an Additional Language, etc.) within the ‘Other’ group was minimal. Due to this, we instead collapsed the ‘Pakistani heritage’ and ‘Other’ group, and adjusted for ethnicity by coding it as 0 = White British, 1 = Other. We chose the White British group as the reference group, as prior literature suggests they may have lower EF and educational achievement overall [3, 16], and it has been recently suggested that the reference category should be chosen such that the presented coefficients are positive [59]. We also conducted a sensitivity analysis including only White British and Pakistani heritage groups (see post-hoc sensitivity analysis).

*Child age.* Child age was recorded in months at the time of taking the Key Stage 2 Assessment and when participating in the BiB executive function tasks. Child age was adjusted for in both models using these variables.

*Child gender.* Child gender was obtained through BiB baseline records and was recorded as 0 = Male, 1 = Female.

*Child English as an Additional Language (EAL).* Child EAL was obtained through national education records and updated annually. It was coded as 0 = Not EAL, 1 = EAL.

### Software

Data cleaning and merging took place in Stata-17, and analysis took place in RStudio (2023.06.0) using the *lavaan* package [56].

### Statistical models

A Structural Equation Model (SEM), which combines measurement models with regression analyses [60], was used for all analyses. Individual measurement models were constructed first for the latent variables (EF, WM, and educational achievement). We constructed two SEMs, and the structure of the models is summarized below (where the first variable is the exposure, second is the mediator, and the last is the outcome): (1) SES → EF/Processing Speed → Educational achievement, and (2) SES → WM/Inhibition → Educational achievement. In addition, all SEMs adjusted for the effects of the covariates described above (child age, child gender, mother immigration status, and child ethnicity). We report standardised estimates for all models, based on the variances of both the observed and latent variables.

### Model fit

For model fit, we report the Comparative Fit Index (CFI) (values > 0.90 are acceptable), Root Mean Square Error of Approximation (RMSEA) (values < 0.07 are acceptable), and Standardised Root Mean Square Residual (SRMR) (values < 0.08 are acceptable) [61].

### Mediation analysis

We conducted two separate SEMs with two mediators included in each, and calculated direct, indirect, and total effects for each model. The direct effect is the pathway from the predictor variable (SES) to the outcome (educational achievement), whilst controlling for the mediator (EF/processing speed or WM/inhibition, depending on the model). The indirect effects describe the pathway from SES to educational achievement through the mediators. Finally, the total effect is the sum of the direct and indirect effects of SES on educational achievement.

All indirect effects are reported using bootstrapped confidence intervals, which is a non-parametric resampling procedure used to assess the variability of a statistic by examining the variability of the sample data [62]. Bootstrap replications were conducted with 1000 repetitions per model. All coefficients are reported using the completely standardised solution (where both observed and latent variables are standardised).

### Missing Data

Little’s MCAR test was used to test the null hypothesis that the missing data is Missing Completely At Random (MCAR) on all variables included in the analysis model [63]. The *p* value (< 0.01) indicated that the missing data is not MCAR (i.e., is either Missing At Random or non-ignorable).

Full Information Maximum Likelihood (FIML) estimates a likelihood function for each individual based on the variables that are present so that all the available data are used, and has been found to produce efficient and unbiased estimates when data are Missing at Random (MAR) [64]. We chose FIML over multiple imputation as FIML appears to perform very similarly to multiple imputation for handling missing data in SEM [65], and it is integrated into the *lavaan* package in R. MAR assumes that the probability that an observation is missing the outcome variable depends on other observed variables in the model, but not on the values of the outcome itself. Since the values of the outcome variables (EF, processing speed, and educational achievement) depend upon child age (i.e., children who are too young to take EF measures are missing them, and children who have not yet reached Key Stage 2 are missing educational achievement), and not on the actual values of those variables (i.e., children with lower EF or educational achievement are not more likely to be missing these variables), data are assumed to be MAR in this analyses.

In the *lavaan* package in R, FIML works by including only those participants who have complete data on exogenous variables in a single model (i.e., all external predictors; SES, Ethnicity, Mother Immigration Status, Child Age, and Child Gender), and estimates associations between the endogenous variables (EF, Processing speed, Educational achievement) even if data are missing/incomplete on any of these variables.

### Post-hoc sensitivity analysis

We conducted three additional post-hoc analyses that were not pre-registered. Due to only a sub-sample of children having the outcome variable available, we conducted analyses examining the same models in children with complete data available on all variables.

Due to modelling constraints, it was also not possible in this study to include a coefficient for > 2 ethnic group(s). Whilst we acknowledge that it may not be appropriate to combine all ethnic minority groups into one group, we wished to retain the smaller ethnic minority groups in the primary analysis. We therefore adjust for ethnicity in the primary analysing by including all ethnic minority groups (0 = White British, and 1 = Other), but also conducted a post hoc sensitivity analysis comparing only the two most populous ethnic minority groups in the cohort (0 = White British, 1 = Pakistani heritage).

To check the sensitivity of the results to our use of a latent class measure of SES, we repeated the analysis with an additional SES indicator. As parental education and occupation are not consistent measures of SES in a multi-ethnic population, we repeated the analysis with the variable ‘how well mother and husband/partner are managing financially’, where responses were coded as 1 = Living Comfortably, 2 = Doing alright, 3 = Just about getting by, 4 = Quite difficult, and 5 = Very difficult.

## Results

### Participants

Figure 2 provides an overview of the availability of measures in the study sample. There were 13,858 individual children enrolled in the BiB cohort. Children with Special Education Needs (SEN) were removed from the sample (*n* = 2210). This left a sample of 11,648 children. As the FIML method only includes participants in the model if they have data on exogenous variables, this resulted in a total of *n* = 4201 children included in the estimation sample. Demographic information for the estimation sample on all variables is provided in Table 1, and correlations between all variables are provided in Table 2.

**Table 1:** Sociodemographic variables for estimation sample (*n* = 4201)

Variables	Frequency (%)
**Age in years at time of executive function measure**	
7	1143 (27)
8	2127 (51)
9	847 (20)
10	84 (2)
Missing	0 (0)
**Age in years at Key Stage 2**	
10	120 (3)
11	681 (16)
Missing	3400 (81)*
**Socioeconomic status**	
Least deprived	683 (16)
Employed, not materially deprived	1372 (32)
Employed, no access to money	683 (16)
Benefits, but coping	749 (18)
Most deprived	714 (17)
Missing	0 (0)
**Child gender**	
Male	1942 (46)
Female	2259 (54)
Missing	0 (0)
**Ethnic group**	
White British	1174 (28)
Other	3027 (72)**
Missing	0 (0)
**Mother immigration status**	
Born in UK	2415 (57)
Born outside UK	1786 (43)
Missing	0 (0)
**English as an Additional Language**	
Yes	2117 (50)
No	2084 (50)
Missing	0 (0)

Note: *=there is high missingness in age in years as not all children had the outcome variable at Key Stage 2, as the entire cohort were yet to reach this age at the time of the data curation. **=within the Other ethnic group, the majority of children were Pakistani heritage (*n* = 2382, 57%), and the next largest group was Indian (*n* = 122, 1%)

We compared the differences between the estimation sample and the whole BiB sample on ethnicity and SES. In the BiB sample, the ethnic group distributions were White British (39%), and ethnic minority groups (61%) [48]. The socioeconomic grouping distributions were “Least deprived” (20%), “Employed and not materially deprived” (19%), “Employed and no access to money” (16%), “Benefit, but coping” (29%) and “Most economically deprived” (16%) [36]. The estimation sample does appear to differ from the BiB sample, in that the Ethnic minority groups and the ‘employed, not materially deprived’ socioeconomic groups are overrepresented.

**Table 2:** Pairwise correlations for variables included in models (estimation sample, *n* = 4201)

Variable (*n*)	Mean	SD	(1)	(2)	(3)	(4)	(5)	(6)	(7)	(8)	(9)	(10)	(11)	(12)	(13)	(14)	(15)
(1) EAL (4201)	**1.50**	**0.50**	1.000														
(2) IMS (4201)	**0.43**	**0.49**	0.474	1.000													
(3) Gender (4201)	**1.54**	**0.50**	0.013	0.001	1.000												
(4) Ethnicity (4201)	**0.28**	**0.49**	-0.620	-0.484	0.010	1.000											
(5) SES (4201)	**1.87**	**1.35**	-0.167	-0.106	-0.054	0.087	1.000										
(6) Age in months (4201)	**101.05**	**8.02**	-0.089	-0.057	0.000	0.131	0.033	1.000									
(7) Age in years (801)	**10.85**	**0.36**	0.052	-0.015	0.059	-0.027	-0.100	0.504	1.000								
(8) FDR (4179)	**0.68**	**0.14**	0.047	0.037	-0.044	-0.090	0.113	0.185	-0.003	1.000							
(9) BDR (4193)	**0.57**	**0.018**	-0.025	-0.014	0.030	-0.002	0.124	0.263	-0.014	0.494	1.000						
(10) Corsi (4164)	**0.59**	**0.17**	-0.022	-0.016	-0.106	-0.012	0.115	0.235	-0.021	0.358	0.445	1.000					
(11) Processing speed (4113)	**5.01**	**1.27**	0.097	0.078	0.065	-0.104	-0.061	-0.289	0.001	-0.173	-0.281	-0.330	1.000				
(12) Inhibition (3955)	**1.33**	**0.58**	0.067	0.037	0.159	-0.045	-0.077	-0.190	0.076	-0.148	-0.220	-0.292	0.413	1.000			
(13) Maths (801)	**106.70**	**5.99**	0.073	0.074	-0.075	-0.041	0.220	0.065	-0.040	0.340	0.423	0.393	-0.242	-0.209	1.000		
(14) Reading (801)	**105.39**	**6.85**	0.040	0.024	0.131	-0.001	0.213	0.087	0.021	0.276	0.291	0.201	-0.117	-0.111	0.584	1.000	
(15) GPS (801)	**108.51**	**6.70**	0.123	0.090	0.160	-0.141	0.181	0.082	0.010	0.347	0.384	0.269	-0.157	-0.130	0.707	0.674	1.000

Abbreviations: BDR = Backwards Digit Recall, EAL = English as an Additional Language, FDR = Forwards Digit Recall, GPS = Grammar, Punctuation and Spelling, IMS = Mother Immigration Status, SES = socioeconomic status. Shaded = *p* < .05

### Analysis

Full analyses and code are available at https://osf.io/p7z6q, and a summary of key results are provided here.

#### Changes from pre-registration

Following the pre-registration several minor changes to the analysis were made. First, we adjusted for ethnicity using only two categories (White British vs. Other) instead of three as planned (White British, Pakistani heritage, Other). This was done because the numbers within the ‘Other’ category were too small to estimate the model. Second, it was not possible to create a new continuous latent measure of SES as planned, as the 17 different variables used were on various scales (dichotomous, categorical, and continuous), and the resulting continuous variable did not appear to present as much variation in SES. We instead used the previously validated measure of socioeconomic position for BiB – a five category variable.

#### Model 1: mediation of executive functions versus processing speed

Model 1 compared EF (modelled as a latent variable) to processing speed (modelled as an observed variable). The model fit values generally indicated adequate fit (CFI = 0.858, RMSEA = 0.066 [0.062 to 0.069], SRMR = 0.044), although the CFI value is slightly lower than is considered acceptable (where < 0.90 is acceptable [61]). The R^2^ value for the latent educational achievement variable was 0.501, showing that the model explains 50% of the variance in this outcome.

**Table 3:** Standardised parameters describing total, direct, and indirect effects for model 1 (*n* = 4201)

	Estimate [95% CI]	*p* value	Mediation ratio
Total effect (combined effect of all below paths)	0.259 [0.195 to 0.322]	< 0.001	100%
Direct effect (SES→educational achievement)	0.152 [0.089 to 0.215]	< 0.001	58%
Bootstrapped indirect effect (SES→executive function→educational achievement)	0.109 [0.082 to 0.135]	< 0.001	42%
Bootstrapped indirect effect (SES→processing speed→educational achievement)	-0.002 [-0.006 to 0.001]	0.397	< 1%

Note: The mediation ratio was calculated by dividing the effect estimate by the ‘total effect’ estimate

**Fig. 3:**
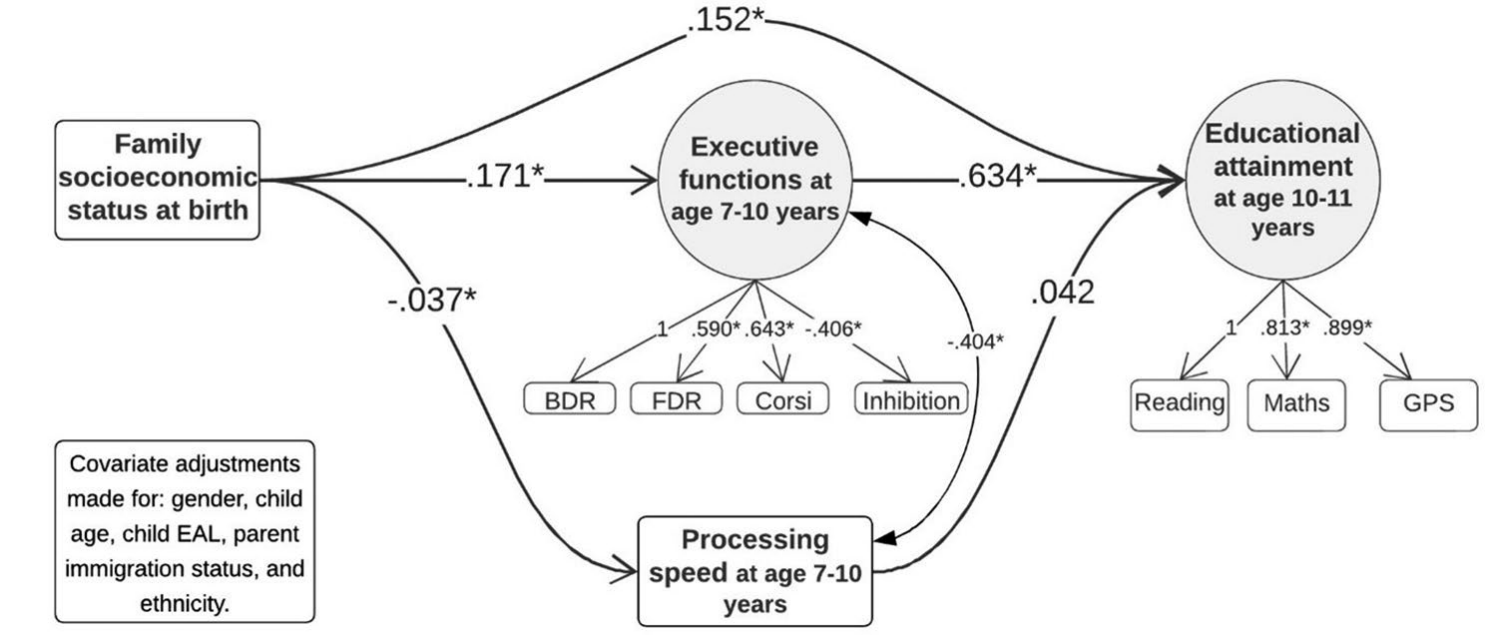
Path diagram of Model 1 presenting standardised coefficients. Note: Covariance between variables and model covariate paths not shown for simplicity of graph. A higher processing speed score is worse ability, hence the negative coefficient. Abbreviations: BDR = Backwards Digit Recall, FDR = Forwards Digit Recall, GPS = Grammar, Punctuation & Spelling, EAL = English as an Additional Language

As seen in Table 3, the total effect estimate was *B* = 0.259 [0.195 to 0.320]. The majority of this was represented in the direct effect between socioeconomic status and educational achievement at *B* = 0.152 [0.089 to 0.215] (mediation ratio = 58%), and the indirect effect between socioeconomic status and educational achievement via executive functions at *B* = 0.109 [0.082 to 0.135] (mediation ratio = 42%). The path between socioeconomic status and educational achievement via processing speed was not significant, at *B*=-0.002 [-0.006 to 0.001]. Figure 3 shows the individual paths between the key variables, showing the significant associations between SES, EF, and educational achievement. Although higher socioeconomic status was associated with better processing speed, processing speed was not associated with educational achievement.

#### Model 2: mediation of executive functions (WM versus inhibition)

Model 2 compared WM (modelled as a latent variable) to inhibition (modelled as an observed variable). The model fit values indicated adequate fit, and were more favourable in this model than Model 1 (CFI = 0.930, RMSEA = 0.047 [0.043 to 0.052], SRMR = 0.033). The R^2^ value for the latent educational achievement variable was 0.485, the model therefore explains 49% of the variance in this outcome.

**Table 4:** Standardised parameters describing total, direct, and indirect effects for model 2 (*n* = 4201)

	Estimate [95% CI]	*p* value	Mediation ratio
Total effect (combined effect of all below paths)	0.258 [0.193 to 0.320]	< 0.001	100%
Direct effect (SES→educational achievement)	0.158 [0.095 to 0.221]	< 0.001	61%
Bootstrapped indirect effect (SES→working memory→ educational achievement)	0.100 [0.076 to 0.125]	< 0.001	39%
Bootstrapped indirect effect (SES→inhibition→educational achievement)	0.001 [-0.003 to 0.006]	0.643	< 1%

Note: Mediation ratio was calculated by dividing the effect estimate by the total effect estimate

**Fig. 4:**
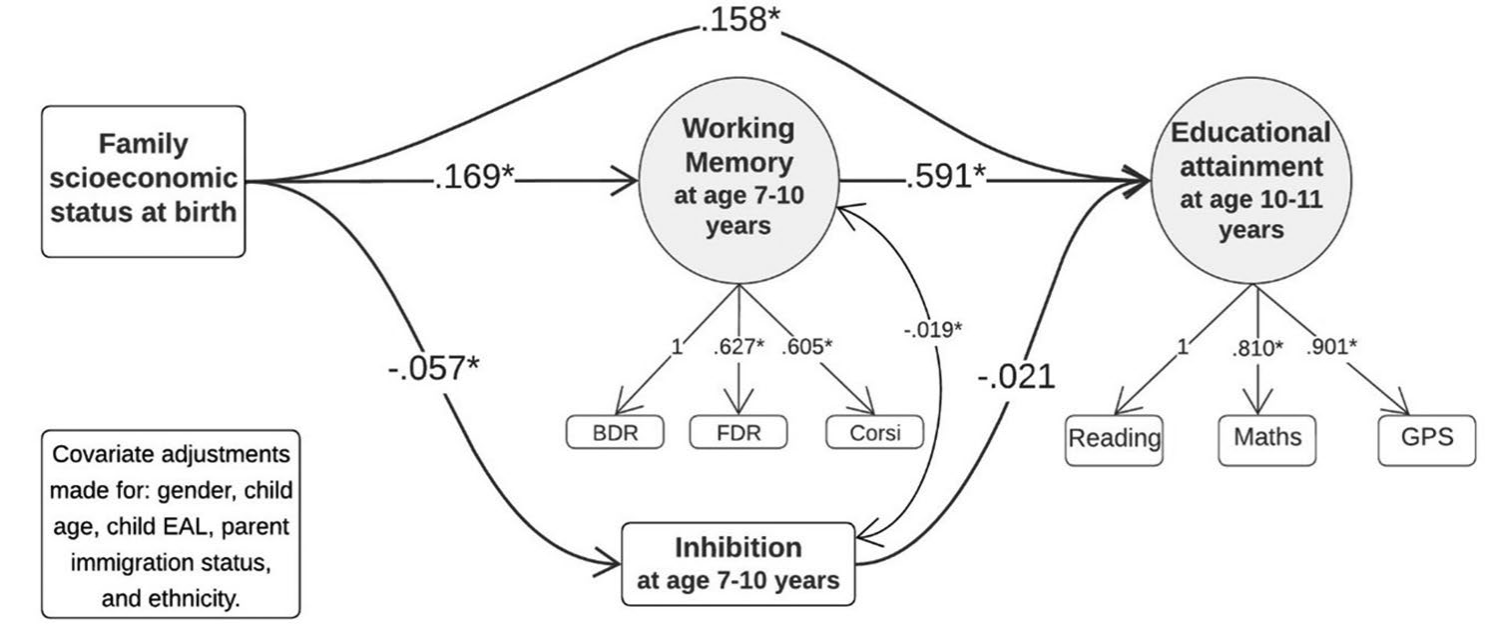
Path diagram of Model 2 representing standardised coefficients. Notes: covariance and model covariate paths not shown for simplicity of graph. A higher inhibition score is worse ability, hence the negative coefficient. Abbreviations: BDR=Backwards Digit Recall, FDR=Forwards Digit Recall, GPS=Grammar, Punctuation & Spelling, EAL = English as an Additional Language

As seen in Table 4, the total effect estimates equalled *B* = 0.258 [0.193 to 0.320]. The majority of this was represented in the direct effect between socioeconomic status and educational achievement at *B* = 0.158 [0.095 to 0.221] (mediation ratio = 61%), and the indirect effect between socioeconomic status and educational achievement via WM at *B* = 0.100 [0.076 to 0.125] (mediation ratio = 39%). The path between socioeconomic status and educational achievement via inhibition was not significant, at *B* = 0.001 [-0.003 to 0.006]. In fact, WM appeared to account for a large proportion (39%) of the total effect, as can be seen by comparing the indirect effect via WM (*B* = 0.100) to the total effect (*B* = 2.58). Figure 4 shows the individual paths between the key variables, showing the significant associations between socioeconomic status, WM, and educational achievement. Although higher socioeconomic status was associated with better inhibition scores, inhibition was not associated with educational achievement.

### Post-hoc sensitivity analysis

The first post-hoc sensitivity analysis to assess the sensitivity of results to missing data with *n* = 734 children revealed the same pattern of results to the primary analysis regarding the direct and indirect effects, though with smaller effect sizes. The second post-hoc sensitivity analysis with only White British and Pakistani ethnic groups also revealed a similar pattern of results to the primary analysis. The third post hoc sensitivity analysis with the different SES indicator (how well mother and husband/partner are managing financially) also revealed a similar pattern of results to the primary analysis. For the full model results and sensitivity analyses, please see https://osf.io/p7z6q.

## Discussion

Using a large, socioeconomically and ethnically diverse sample, this study examined potential mediating pathways in relation to the large and persistent social inequalities in children’s educational outcomes [2, 3]. Our study used data from a cohort that is ethnically diverse, allowing us to adjust for the effects of ethnicity on the mediating and outcome variables. This is important given that ethnicity may be associated with both executive functions and educational achievement [3, 16, 37], and yet is something that has not been consistently addressed in the previous literature. We measured SES just before the child was born, capturing the relations between socioeconomic deprivation in pregnancy and long-term cognitive and educational outcomes. In addition, we measured educational achievement via children’s performance on national tests that become part of the child’s official education record, and therefore have a strong influence on other life outcomes [4], rather than using proxy measures of educational outcomes.

To the best of our knowledge, this is the first study to compare the separable contributions of EF and processing speed within the association between SES and educational achievement. In line with our predictions, we found that a composite measure of executive function significantly mediated the relation between socioeconomic status and educational achievement, supporting the idea that EF is a critical component in understanding the mechanisms by which socioeconomic disadvantage has a detrimental effect on educational outcomes [6, 15, 27]. Given the lack of previous research, we made no predictions about whether processing speed would be a stronger or weaker mediator compared to EF. This current study is the first to identify that children’s processing speed does not significantly mediate the relation between SES and educational achievement. This does not support the idea that differences in processing speed underlie the association between EF and educational achievement [40], and instead suggests that EF is the more important factor when considering educational outcomes.

When considering the direct associations, we found that higher SES was associated with higher EF scores, and that higher EF scores were associated with better educational achievement, supporting previous research in this area [15, 16, 24, 25]. These results underline the importance of children’s very early environments for shaping their later cognitive and educational achievement [34]. This association may have occurred through the impacts of maternal prenatal stress and low nutrition (induced by low SES) upon child cognitive development [17, 18], or through enriched socioeconomic resources providing additional opportunities for EF development [20, 21].

With regards to processing speed, we found that higher SES meant faster processing speed in childhood, supporting the small number of existing studies on this issue [43, 44]. Again, this could be due to the negative impacts of low SES, potentially through chaotic homes [66], or through enriched socioeconomic resources gained from high SES [20]. However, in contrast to previous studies [41, 42], we found no association between processing speed and children’s educational achievement. One possible explanation for this is that the previous studies used two different measures of processing speed simultaneously, and this may have captured more variation in processing speed ability [41, 42]. Another possible explanation for these conflicting findings could be the age and developmental abilities of previous samples. For example, Passolunghi & Lanfranchi (2012) assessed processing speed at 5-years-old and achievement at 6-years-old, and Mulder et al. (2010) assessed children at 9-10-years-old who were born prematurely (and therefore at risk of cognitive impairments) [41, 42]. Our study measured processing speed at 8-years-old and educational achievement at 10-years-old and excluded children with any SEND. It is therefore possible that processing speed may be more influential in determining educational achievement for younger, or more cognitively impaired, children, but becomes less influential for older children. This could be tested further in future studies, by collecting repeated measurements of EF and processing speed, to explore whether the contributions of these change as children grow older.

We also investigated which of the EF components might be driving the association between SES and educational achievement. We found that WM was a strong and consistent mediator, accounting for a large proportion of the variance in the total effect (39%), whereas inhibition was not significant (accounting for < 1% of the variance). To the best of our knowledge, this is the first large-scale, pre-registered study to provide an understanding of these associations at a more granular level, and points towards WM, rather than inhibition, being an important factor in shaping the association between SES and educational achievement.

Indeed, from a theoretical perspective, we can see how difficulties with WM lead to poorer educational outcomes. WM represents the ability to store and manipulate information necessary for the successful completion of the task-at-hand [10], and as such underpins learning [24, 25]. In mathematics, children are required to hold number combinations, and to update the contents of WM to include intermediate steps, in order to complete the task [67]. In reading, WM is needed to keep relevant speech sounds in mind, match them up with corresponding letters, and combine them to read words [68]. WM deficits can therefore result in disruption to learning opportunities in the classroom, which in turn affects academic success and often leads to disengagement [69].

The results from the present study, and possible underpinning theoretical mechanisms, point to the importance of finding ways to support children with WM deficits within the classroom. Historically, research has looked at interventions that sought to improve children’s WM ability via WM training [70]. However, meta-analyses have found no evidence of any clear transfer effects from WM training to improvements in educational achievement [71]. Alternative ways to support children with WM difficulties therefore need to be investigated. A recent survey on WM with nearly 1500 teaching professionals suggests that any intervention should include some element of teacher training [69]. The survey showed that there was considerable variation in teachers’ understanding of WM, with most overestimating the capacity limits of WM, and many unable to list signs of, or strategies to support, WM difficulties [69]. Indeed, further, robust research is needed on the best ways to support children with WM deficits as research to date is limited [72].

## Limitations and implications for future research

There are limitations to the current study that should be considered when discussing recommendations and future research. Whilst the sample included in this study was large relative to other studies (*n* = 4201), only some of these children (20%) had the educational achievement outcome (see Table 1). This is unlikely to have biased the effect estimates provided in this study, since the mechanism used to address missing data, FIML, provides unbiased estimates in the presence of Missing at Random (MAR) data [64], and the numbers for this type of analysis are sufficient [73]. We also conducted a sensitivity analysis including only children with complete data available, and the pattern of results was the same. In addition, although the cohort in this study reflects key aspects of many major cities - areas with high levels of deprivation and communities containing different ethnic groups – the population in this study was drawn from one city within the UK. It is also important to note that our estimation sample does differ from the original BiB cohort in terms of ethnic and socioeconomic groups, though our sample are more ethnically diverse.

Further, this study used observational data with data collection at three separate timepoints (before birth, age 7–10, and age 10–11). Although the longitudinal trajectory of data collection, and the control of several key confounding variables (e.g. ethnicity and mother immigration status), meant that mediation modelling could take place with more confidence than with cross-sectional data [33], we need to exercise caution when discussing any causal nature of the pathways identified in our models. When considering future research, it would be beneficial to include repeated measurements of these constructs to establish their growth and importance over time. Further, establishing causality via randomised studies is challenging given we clearly cannot randomise children to lower or higher socioeconomic positions. However, an ongoing RCT is testing the effects of regular cash payments on children’s outcomes [74]. The trial is in the early stages, but initial results suggest that the payments are having some effect on early developmental differences, and the study will go on to examine if this translates into differences in neurocognitive abilities, including a measure of EF [74].

Of note is that this study is restricted to the variables measured within the BiB cohort study. Whilst there were three separate measures of WM, there was only one measure of inhibition and one measure of processing speed. It is therefore possible that the more reliable measurement of WM (as compared to one task measuring each processing speed and inhibition) has resulted in a larger effect for WM in this study.

Finally, whilst a strength of this study is that it was able to consider socioeconomic differences in these variables across both White British and Pakistani heritage groups, it was beyond the scope of this study to examine whether associations varied *within* these groups. Relationships between SES and various outcomes, including WM, may be stronger within White British ethnic groups (in comparison to Pakistani heritage groups) in the BiB cohort [16, 52]. Whilst this study found a general relationship between SES, WM, and educational achievement across diverse ethnic groups, the relationships may also be more or less prominent within particular ethnic groups, and this is yet to be tested.

## Conclusions

This large, ethnically diverse study examined the roles of different components of EF and processing speed in explaining the relation between early life SES and educational achievement. These results showed that WM was the key mediating factor, with no evidence that processing speed and inhibition contributed to the mediation pathways. This study therefore suggests that supporting WM in the classroom is an important avenue for future research in educational practice, and may be particularly useful for closing the socioeconomic gap in educational achievement.

## Electronic supplementary material

Below is the link to the electronic supplementary material.

Supplementary Material 1

Supplementary Material 2

## Data Availability

These data cannot be shared publicly as they are available through a system of managed open access (see https://borninbradford.nhs.uk/research/how-to-access-data/). This study was preregistered, and analysis code are available at https://osf.io/2v6rz/.
