# Supplementary material for: Do executive functions and processing speed mediate the relationship between socioeconomic status and educational achievement? Analysis of an observational birth cohort study
Source: BMC Psychol. 2024 Dec 18;12:746. doi: 10.1186/s40359-024-02243-1 (PMC11657664; doi:10.1186/s40359-024-02243-1)
Supplement: Supplementary file 1 — Supplementary Material 1 [file 40359_2024_2243_MOESM1_ESM.docx]

## File A: Description of latent socioeconomic groups

| **Class** | **Description** |
| --- | --- |
| Least socioeconomically deprived and most educated” | Women currently and previously employed Father non-manual employment Women and fathers highly educated Up to date with bills Mortgage Not subjectively poor Not receiving means tested benefits Not materially deprived |
| “Employed, not materially deprived” | Women currently employed Father manual and non-manual employment Women and father medium levels of education Up to date with bills Mortgage Not subjectively poor Not receiving means tested benefits Not materially deprived |
| “Employed, no access to money” | Women currently and previously employed Father manual and non-manual employment Women and father’s medium levels of education Moderate behind with bills Mortgage and private renting Moderate subjective poverty Moderate receipt of means tested benefits Materially deprived in particular can’t afford holidays, money to replace goods and savings |
| “Benefits and not materially deprived” | Women low current employment Father manual employment and self-employed Women and father’s low levels of education, father’s education high don’t know response Up to date with bills Owns house outright Not subjectively poor High receipt of means tested benefits Not materially deprived |
| “Most economically deprived” | Women low current employment Father manual employment and unemployed Women and father’s low levels of education, father’s education high don’t know response Behind with bills Private renting and social housing Subjectively poor Highest receipt of means tested benefits Materially deprived |
